# Peduncular Hallucinosis: Clinical characteristics, etiology, and a case report

**DOI:** 10.1192/j.eurpsy.2024.549

**Published:** 2024-08-27

**Authors:** J. Garde Gonzalez, A. Oliva Lozano, P. Herrero Ortega, M. A. Morillas Romerosa

**Affiliations:** Psychiatry, La Paz University Hospital, Madrid, Spain

## Abstract

**Introduction:**

Visual hallucinations are a relatively common neurological complaint. Peduncular hallucinosis (PH) stands out as a distinct entity, characterized by complex visual hallucinations resulting from structural lesions in the brainstem or diencephalon.

**Objectives:**

We aim to provide an overview of the clinical features, etiological factors, and management strategies associated with PH, incorporating a unique case study.

**Methods:**

Clinical case report and brief literature review.

**Results:**

**Clinical Characteristics:** PH is marked by detailed, colorful, vivid, and occasionally emotionally charged visual hallucinations. These hallucinations encompass people, animals, or objects and may be mistaken for reality. While primarily visual, they may occasionally involve other sensory modalities. Crucially, patients with PH maintain insight, distinguishing it from primary psychotic disorders.

**Etiological Factors:** PH is most commonly associated with structural brainstem lesions, particularly in the midbrain. Potential instigators encompass ischemic strokes, vascular anomalies, tumors and infections. Disruption of the reticular activating system in the brainstem is implicated in the pathogenesis. Advanced imaging techniques have unveiled cases with subtle presentations, broadening our comprehension of PH.

**Pathogenesis:** PH may involve the disturbance of serotonergic inhibitory pathways and the reticular activating system. A plausible link with hypnagogic hallucinations hints at a mechanism related to rapid REM sleep transitions.

**Case Report:** Mr. J., a 30-year-old patient, suffered recurrent mesencephalic strokes attributed to Sneddon’s syndrome, ADA-2 deficiency, and protein C deficiency, leading to malacic lesions in the hemimesencephalon and right hemipons. He experienced complex visual hallucinations, primarily geometric patterns and animals, mainly at night. Importantly, he maintained insight into their hallucinatory nature. Mr. J. also had diplopia, visual impairment, recurrent headaches, and left hemiparesis.

Reactive anxiety and depression due to functional loss followed his recurrent strokes. Initially, antipsychotics were used to manage sensory-perceptual disturbances, but were later discontinued due to reduced interference with daily functioning. Antidepressant and psychological therapy was continued throughout the follow-up to address mood symptoms.

**Conclusions:**

Peduncular hallucinosis is an intriguing phenomenon characterized by complex visual hallucinations. Understanding its clinical features, etiology, and possible mechanisms is essential for accurate diagnosis and management. This case report emphasizes PH’s clinical aspects and the importance of a multidisciplinary approach, including pharmacological intervention and psychological support. Understanding its features, causes, and management is essential for accurate care. Further research is needed to improve our comprehension and optimize treatment strategies.

**Disclosure of Interest:**

None Declared

